# Cleft lip and palate transmembrane protein 1-like is a putative regulator of tumorigenesis and sensitization of cervical cancer cells to cisplatin

**DOI:** 10.3389/fonc.2024.1440906

**Published:** 2024-09-13

**Authors:** Weipeng Liu, Fengdan Huang, Yueting Yao, Yan Liang, Zhiling Yan, Lili Guo, Xinwen Zhang, Li Shi, Yufeng Yao

**Affiliations:** ^1^ Institute of Medical Biology, Chinese Academy of Medical Sciences and Peking Union Medical College, Kunming, Yunnan, China; ^2^ Graduate School of Yunnan University, Yunnan University, Kunming, Yunnan, China; ^3^ College of Nursing Health Sciences, Yunnan Open University, Kunming, Yunnan, China; ^4^ Department of Gynaecologic Oncology, Peking University Cancer Hospital Yunnan & Yunnan Cancer Hospital & The Third Affiliated Hospital of Kunming Medical University, Kunming, Yunnan, China

**Keywords:** CLPTM1L, cervical cancer, proliferation, apoptosis, cisplatin

## Abstract

**Background:**

Cervical cancer stands as one of the leading causes of cancer-related mortality in women worldwide, yet the precise functions of host genes implicated in its pathogenesis remain elusive. Genome-wide association studies (GWAS) have revealed a significant association between the CLPTM1L locus and cervical cancer risk in European women, and aberrant expression of CLPTM1L has been noted in various malignant tumors. However, the role of CLPTM1L in cervical cancer remains largely unexplored.

**Methods:**

The expression of CLPTM1L in cervical cancer cells and tissues was detected by RT-qPCR. Furthermore, the potential biological functions of CLPTM1L in the context of cervical cancer were explored via RNA sequencing. Cell proliferation rates and the responsiveness of cervical cancer cells to cisplatin were evaluated using the CCK-8 assay, while cell apoptosis was quantified through the utilization of flow cytometry. Nude mouse xenograft models were utilized to explore the impact of CLPTM1L on tumor formation *in vivo*.

**Results:**

Our findings demonstrated a significant increase in CLPTM1L mRNA expression levels in HeLa and C33A cells, as well as in cervical carcinoma tissues, compared to ECT1/E6E7 cells and adjacent normal tissues. Genes related to CLPTM1L were found to be enriched in the Hedgehog signaling pathway. *In vitro* and *in vivo* studies showed that reducing CLPTM1L expression markedly inhibited cell proliferation via downstream candidate genes BOC and LRP2. Furthermore, the downregulation of CLPTM1L was found to enhance cisplatin-induced cell apoptosis and increase the susceptibility of cervical cancer cells to cisplatin through DAP1.

**Conclusions:**

CLPTM1L could impact cervical cancer cell proliferation and cisplatin-induced cell apoptosis, as well as cisplatin susceptibility in cervical cancer cells. This investigation has bestowed upon us novel insights into the pathogenesis of cervical cancer, underscoring the potential of CLPTM1L as a promising target for chemotherapeutic sensitization in the management of this malignancy.

## Introduction

Cervical cancer ranks as the fourth most prevalent malignant neoplasm affecting women’s health globally. In 2020, there were over 600,000 reported new cases of cervical cancer and about 340,000 deaths worldwide (1). In the reference scenario, the number of global cervical cancer-related DALYs (disability-adjusted life-years) is projected to increase from 10 million DALYs in 2022 to 12. 4 million DALYs in 2050 (2). Persistent infection with high-risk strains of the human papillomavirus (HPV) stands as the primary risk factor for cervical cancer (3–6). While the majority of women infected with HPV can clear the infection naturally, only a small fraction will experience persistent infection, potentially leading to the development of cervical carcinoma (7, 8). This observation suggests that host genetic factors may exert a considerable influence on cervical cancer risk (9–11).

Chemotherapy remains the cornerstone of treatment for cervical cancer patients (12). Cisplatin, a diminutive platinum-based compound, stands as the quintessential therapeutic agent for addressing locally advanced or recurrent cervical cancers (13–15). Nonetheless, the emergence of drug resistance phenomena poses a formidable challenge, potentially curtailing the life expectancy of cervical cancer patients (16, 17). Consequently, the imperative to discern efficacious molecular targets to circumvent this obstacle in cervical cancer treatment becomes undeniable.

Recent genome-wide association studies have elucidated a correlation between genetic variation in CLPTM1L at 5p15.33 and the susceptibility to cervical cancer (18, 19). In 2023, Sara et al. further pinpointed a genome-wide significant locus adjacent to CLPTM1L in the context of cervical dysplasia (20). CLPTM1L has been identified as one of the foremost candidate genes for cervical cancer (20). Encoding a transmembrane protein, CLPTM1L exhibits heightened expression across various malignant tissues, including the lung lung (21), ovary (22), pancreas (23, 24) and Oral Squamous Cell Carcinoma (25). Research indicates that CLPTM1L fosters tumor proliferation in pancreatic cancer (23) and oral squamous cell carcinoma (25). Puskás et al. demonstrated that monoclonal antibodies targeting CLPTM1L effectively suppress the growth of lung and pancreatic cells both *in vitro* and *in vivo* (26). Earlier investigations have associated CLPTM1L with cisplatin-induced apoptosis in ovarian tumor cells (27). Furthermore, another study revealed that knockdown of CLPTM1L enhances cisplatin-induced apoptosis in lung cancer cells (28). RNAi-mediated knockdown of CLPTM1L increased chemosensitivity to cisplatin in human lung cancer 95-D cells and cisplatin-induced activation of caspase-9 and caspase-3/7 (29). Li et al. reported that CLPTM1L directly interacts with and acts as a coactivator of the transcription factor ERβ, leading to the activation of ERβ target genes in NSCLC cells and thereby promoting radio resistance (30). These studies indicate that CLPTM1L can further participate in the occurrence and development of various cancers by influencing cell proliferation, apoptosis, as well as chemotherapy drug resistance. Nevertheless, the expression patterns and roles of CLPTM1L in the pathogenesis and therapeutic strategies of cervical cancer await comprehensive elucidation.

The current study aims to scrutinize the expression of CLPTM1L in cervical cancer tissues and cells, delving deeper into its potential biological role in the pathogenesis of cervical cancer and its impact on the sensitivity of cervical cancer cells to cisplatin.

## Materials and methods

### Tissues

This study received approval from the Ethics Committee of the Third Affiliated Hospital of Kunming Medical University (approval no. KYCS2021232), and written informed consent was obtained from all participants. A total of 95 matched sets of primary cervical cancer tumors and adjacent normal tissues were obtained from patients at the Third Affiliated Hospital of Kunming Medical University. Tumor tissues and adjacent normal tissues were collected by the surgeon immediately after tumor resection. The adjacent normal tissues were obtained from a segment located more than 2 cm away from the tumor edge, as per the clinician’s visual assessment and clinical expertise. The patient demography including the FIGO stages and histologic types of primary cervical cancer tissues is provided in **Table 1**. The 95 patients aged between 22 and 68 years old, with an average age of 47.01 ± 10.46 (mean ± SD) years (**Table 1**). The patients did not undergo any treatments such as radiotherapy, chemotherapy, targeted therapy, or any other interventions that could potentially impact the tumor. Additionally, the patients did not have any other tumors or systemic diseases. All tissues underwent pathological examination and were promptly frozen at -80°C.

**Table 1:** Demographic characters of cervical cancer patients.

	Cervical cancer patients
**Number**	95
**Ages**	47.01 ± 10.46
FIGO stages	
I	55
II	15
III	25
IV	0
Histologic types	
Adenocarcinoma	19
Squamous cell carcinoma	71
Others	5

### Reverse transcription-quantitative PCR

Total RNA was extracted from tissues or cells utilizing TRIzol reagent. Subsequently, the PrimeScript™ RT reagent Kit with gDNA Eraser (cat. no. RR047A; Takara Bio Inc.) was employed for cDNA synthesis. We reverse transcribed 1 µg of total RNA and diluted the cDNA to a final concentration of 20 µg/µL. Quantitative real-time PCR was conducted under the following conditions, employing GoTaq^®^qPCR Master Mix(cat. no. A6002; Promega): initial denaturation at 95°C for 10 min, followed by 40 cycles of denaturation at 95°C for 15 s, annealing at 60°C for 15 s, and extension at 72°C for 15 s. Relative mRNA expression levels were determined using the 2^-ΔΔCq^ method (31) and β-actin served as the internal control for CLPTM1L. The specific primer pairs of PCR were designed using the Primer Premier 5 (Premier Biosoft International, Palo Alto, CA, USA) program according to the exon sequence of target genes. The Whitehead siRNA (short interfering RNA) Selection Web Server (32) (http://jura.wi.mit.edu/bioc/siRNA) was used for designing siRNA of target genes. Details of all primers and shRNA target sequences utilized are provided in **Table 2**.

**Table 2:** List of primers and shRNA target sequences used.

Name	Sequence (5’- 3’)	RefSeq
β-actin-F	TGCGTGACATTAAGGAGAAGC	NM_001101.5
β-actin-R	TCCATGCCCAGGAAGGAA	
CLPTM1L-F	GGGTGCTGAGAACAACATCG	NM_030782.5
CLPTM1L-R	GCGTCCCATTGTTTCTCGTT	
BOC-F	CCTCCAAGGATGAATGTAACCTG	NM_001378074.1
BOC-R	GAGATTGGCTAGTGTCACAGTG	
LRP2-F	GTTCAGATGACGCGGATGAAA	NM_004525.3
LRP2-R	TCACAGTCTTGATCTTGGTCACA	
DAP1-F	TCATCTCTGGGGTCATCGCC	NM_004394.3
DAP1-R	GGGTTCTTGGGGAAGGATGC	
SEC24D-F	ACCCACCACACTCAATGGTC	NM_014822.4
SEC24D-R	GTAAGACAGTTGTGCGCCTG	
MUC13-F	GATCCCTGTGCAGATAATTCGTT	NM_033049.4
MUC13-R	ACTATGCAAGTCTTGATAGGCCA	
CBX6-F	GAGGGGACCCAAACCCAAAA	NM_014292.5
CBX6-R	ATGTCCTTCTTGAGCCGGTG	
MAMDC2-F	TGAACCGCTGGAATCCCAAT	NM_153267.5
MAMDC2-R	CAACGGGGAGATGAGCTGTG	
FMOD-F	GAGACCTACGAGCCTTACCC	NM_002023.5
FMOD-R	TTGAGGTTGCGATTGTCACAG	
CLPTM1L-shRNA1	GCCAGAAGAAATCAACCTG	NM_030782.5
CLPTM1L-shRNA2	GATGCTGATGAGGTGAAAG	
LRP2-shRNA1	GTGGACATTGTGTACACAG	NM_004525.3
LRP2-shRNA2	GGAGGATTTATCTGCTCCT	
BOC-shRNA1	GGATGAATGTAACCTGGCG	NM_001378074.1
BOC-shRNA2	GTTAGATGTGCAGCACGTG	
DAP1-shRNA1	GACAAGGATGACCAGGAAT	NM_004394.3
DAP1-shRNA2	GCATGCATCTTAGAAATAG	

### Western blotting

Cells were rinsed with phosphate-buffered solution (PBS), then lysed in RIPA and cOmplete™ protease inhibitor cocktail (cat. no. 4693159001; Roche) mixed lysis buffer for 30 minutes, followed by concentration under 12,000 x g at 4°C for 10 minutes. Protein concentration was determined using Pierce™ BCA Protein Assay Kits (cat. no. 23227; Thermo Scientific). Subsequently, 50 μg of total protein was loaded into each lane and subjected to electrophoresis using 12% SDS-PAGE, followed by transfer onto PVDF membranes (cat. no. IPVH00010; MilliporeSigma). The membranes were then blocked with 5% non-fat milk for 2 hours at room temperature and subsequently incubated overnight with anti-CLPTM1L antibody (1:1,000; cat. no. ab256451; Abcam) and anti-GAPDH antibody (1:2,000; cat. no. ab181602; Abcam), followed by incubation with secondary antibodies (1:5,000; cat. no. 7074; Cell Signaling) for 1 hour. Membranes were washed with TBST buffer and then exposed to Clarity™ Western ECL Substrate (cat. no. 1705060; Bio-Rad) for 3 minutes before visualization.

### Cell culture

Ect1/E6E7 was originally obtained from the American Type Culture Collection (ATCC). HeLa and C-33A utilized in this study were procured from the Servicebio Biotechnology Co., Ltd. The cells were carefully examined using short-tandem repeat profiling to verify their authenticity and to guarantee that they were not contaminated. Ect1/E6E7, HeLa, and C-33A cells were maintained in Dulbecco’s Modified Eagle medium (DMEM; cat. no. C11965500BT; Gibco) supplemented with 10% fetal bovine serum (FBS; cat. no. 10100147C; Gibco). Cultivation of all cell lines took place in an incubator at 37°C with 5% CO2.

### The establishment of stable cell lines with targeted gene knockdown

The lentiviral vector pSicoR-Ef1a-mCh-Puro was employed to attenuate the expression of target gene in HeLa and C-33A cells. Two distinct shRNA sequences were designed for each target gene. These shRNA sequences were integrated into the vector and validated through sequencing. Lentiviral constructs were then utilized to infect HeLa cells for a duration of 72 hours. Following this, cells underwent screening in medium supplemented with 2 µg/mL puromycin (cat. no. P8230; Solarbio Life Sciences) for a period of 2 weeks. Following the screening period, flow cytometric analysis of mCherry-expressing cells was employed to assess the infection efficiency of stably infected cells. Subsequently, the expression levels of CLPTM1L were confirmed via RT-qPCR and Western blot analysis. The specific shRNA sequences utilized are detailed in **Table 2**.

### RNA-sequence analysis

Total RNA was extracted from a total of 9 samples, comprising three negative control samples, three samples infected with pSicoR-Ef1a-mCh-Puro-CLPTM1L-shRNA1, and three samples infected with pSicoR-Ef1a-mCh-Puro-CLPTM1L-shRNA2. Subsequently, a cDNA library was constructed and sequenced using the BGISEQ-500 platform (MGI Tech; China). Transcriptome data analyses were conducted using an online platform (https://biosys.bgi.com).

### Cell counting kit-8 assay

The Cell Counting Kit-8 (CCK-8) assay (cat. no. GK10001; GLPBIO) was employed to assess cell proliferation as previously described (33). Briefly, cells were seeded into 96-well plates and incubated at 37°C with 5% CO2 for 3 days. At specified time intervals (0 h, 24 h, 48 h, and 72 h), 10 µl of CCK-8 reagent was added to each well and incubated at 37°C for 1 hour. Absorbance was then measured at 450 nm, and a growth curve was constructed based on the absorbance readings. The blank control group was set up as follows: CCK8 was added to the cell-free medium, incubated for a specific period, and the absorbance at 450 nm was measured as a blank.

### Cell apoptosis assay

Cell apoptosis was assessed using the FITC Annexin V Apoptosis Detection Kit with 7-AAD (cat. no. 640922; BioLegend) as previously described (34, 35). Cells were rinsed twice with cold PBS and subsequently resuspended in 1X Binding Buffer at a concentration of 1 x 10^6 cells/mL. Next, 100 µL of the solution (equivalent to 1 x 10^5 cells) was transferred to a 5 mL culture tube. Following this, 5 µL of FITC Annexin V and 5 µL of 7-AAD were added. The cells were gently vortexed and then incubated for 15 minutes at room temperature (25°C) in the absence of light. Finally, 400 µL of 1X Binding Buffer was added to each tube, and the samples were promptly analyzed by flow cytometry within 1 hour.

### Chemosensitivity assay

Cells were seeded into 96-well plates and allowed to adhere for 24 hours. Subsequently, cells in each group were treated with varying concentrations (0, 1, 2.5, 5, 10, 20, 40, 60, and 80 μmol/L) of cisplatin(cat. no. IC0440; Solarbio Life Sciences). Following a 24-hour incubation period, 10 μL of CCK-8 solution was added to each well. The cells were then further incubated at 37°C for 1 hour, after which the optical density (OD) value was measured at 450 nm. The half-inhibitory concentration (IC50) was determined by plotting the growth inhibitory rate curve for each group.

### Tumor xenograft assay

Six-week-old female BALB/c-nude mice were acquired and housed in a specific pathogen-free environment. A total of 1x10^6 HeLa cells suspended in 100 μL of PBS were subcutaneously inoculated into the right hind groin of nude mice (6 animals per experimental group). Tumor volumes were measured twice a week, and tumor volume was calculated using the following formula: Volume (mm^3^) = length × width^2/2. On day 23 post-inoculation, the mice were euthanized, and final tumor volumes were assessed.

### Statistical analysis

The data in the present study are presented as mean ± standard deviation for continuous variables and were analyzed using GraphPad Prism 8.3.0 software. Each experiment was conducted at least three times. Pairwise or unpaired Student’s t-tests were employed for comparisons between two groups. One-way ANOVA with Tukey’s multiple comparison test was utilized for multi-group comparisons. P<0.05 was considered to indicate a statistically significant difference.

## Results

### CLPTM1L expression was increased in cervical cancer cells and tissues

**Figure 1:**
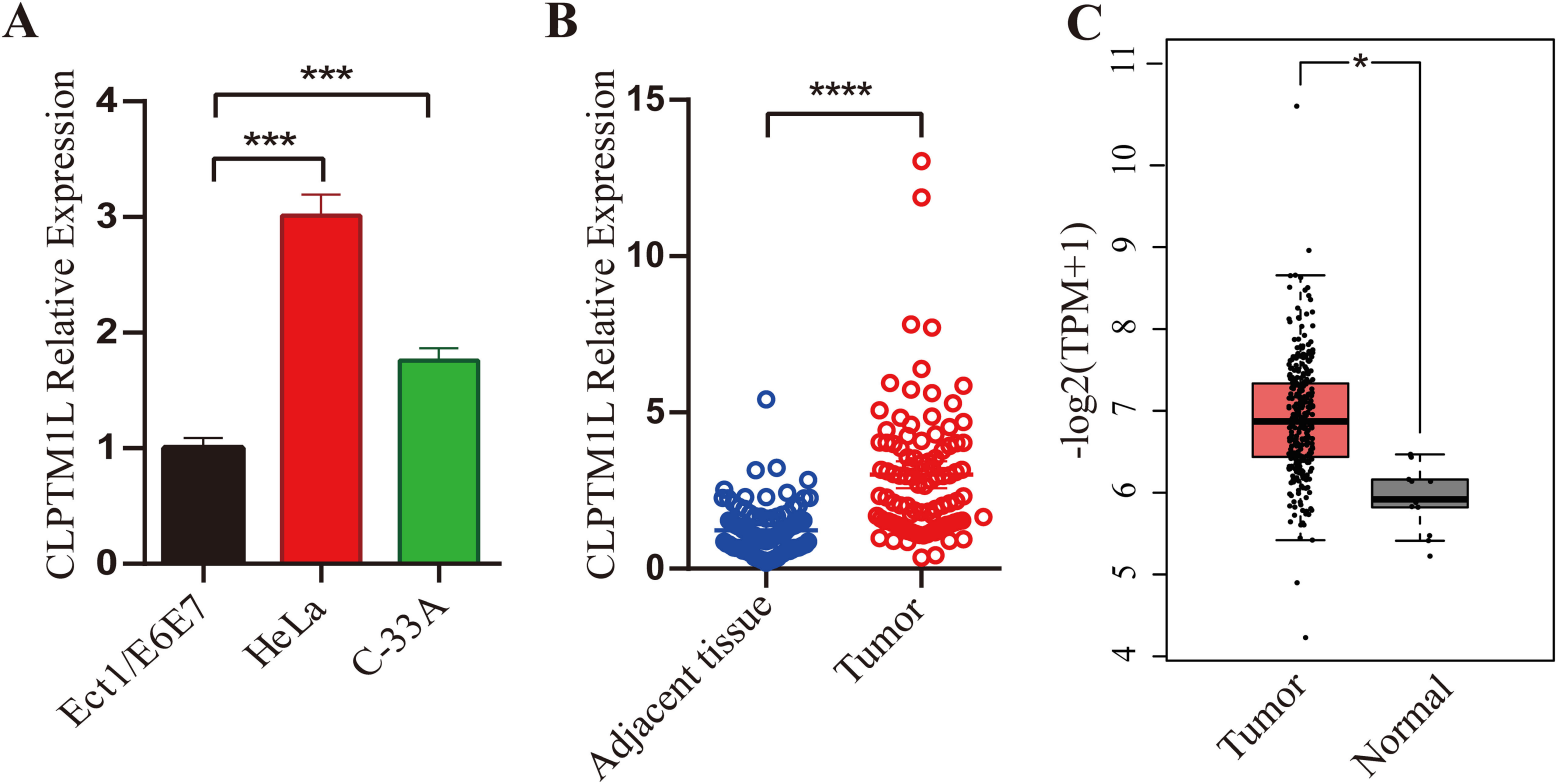
Expression of CLPTM1L in cervical cancer cells and tissues. **(A)** CLPTM1L mRNA expression was measured by qRT-PCR in ECT1/E6E7, HeLa, and C-33A cells. The expression levels of CLPTM1L were normalized to beta-actin levels. **(B)** CLPTM1L mRNA expression was examined via qRT-PCR in 95 cervical cancer tissues and their matched noncancerous tissues. CLPTM1L expression levels were normalized to beta-actin levels. Data were compared using a two-tailed paired t-test. **(C)** Relative expression levels of CLPTM1L in cervical cancer tissue and normal tissue were obtained from the GEPIA database. *P<0.05, ***P<0.001, ****P<0.0001.

qRT-PCR was carried out to reveal the expression of CLPTM1L mRNA in cervical cancer cells and tissues. The findings revealed a significant elevation in CLPTM1L expression in cervical cancer cells (HeLa and C-33A) compared to Ect1/E6E7 cells (**Figure 1A**). Subsequently, mRNA levels of CLPTM1L were examined in 95 cervical cancer tissues along with their matched noncancerous tissues. As depicted in **Figure 1B**, CLPTM1L expression exhibited a marked increase in cervical cancer tumor tissues compared to normal cervical tissues. This observation was further validated by data from the Gene Expression Profiling Interactive Analysis (GEPIA) database (36) (**Figure 1C**). Collectively, these results suggest that aberrant expression of CLPTM1L may play roles in cervical cancer pathogenesis.

### Lentivirus-mediated CLPTM1L silencing in HeLa and C-33A cells

**Figure 2:**
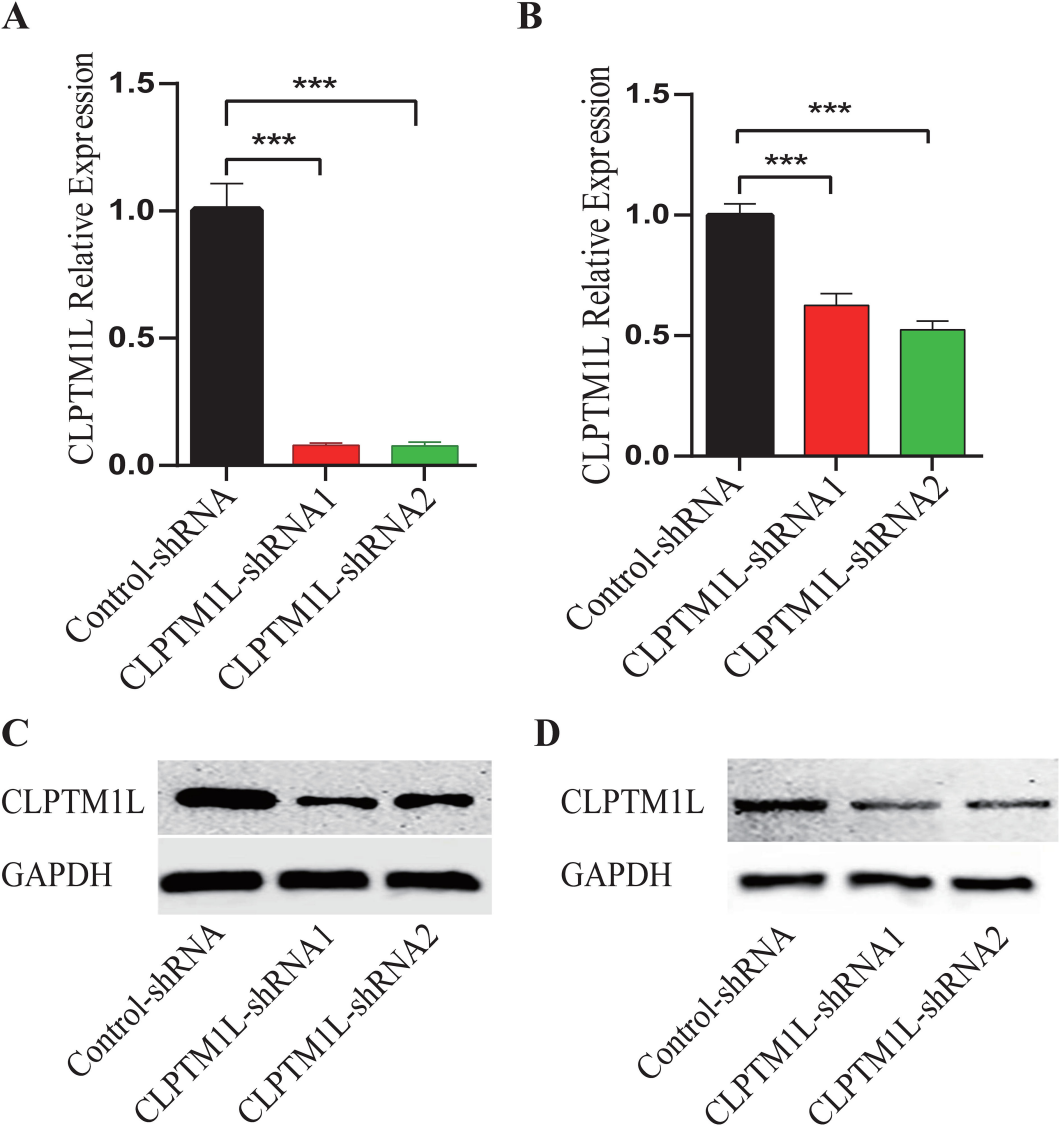
Construction of CLPTM1L knockdown HeLa and C-33A cell lines. **(A)** The knockdown expression of CLPTM1L was confirmed via RT-qPCR in HeLa. **(B)** The knockdown expression of CLPTM1L was confirmed via RT-qPCR in C-33A. **(C)** The knockdown expression of CLPTM1L was confirmed via Western blot in HeLa. **(D)** The knockdown expression of CLPTM1L was confirmed via Western blot in C-33A. ***P<0.001.

To investigate the roles of CLPTM1L in cervical cancer cells, lentiviral vectors containing shRNA sequences designed to specifically silence the expression of CLPTM1L were constructed for infection of HeLa and C-33A cells. Flow cytometric analysis revealed that more than 80% of the infected cells in each group exhibited positivity for mCherry, indicating successful infection (**Supplementary Figure S1** and **Supplementary Figure S2**). Real-time PCR analysis demonstrated a notable reduction in CLPTM1L mRNA expression in cells infected with CLPTM1L-shRNA compared to the control group (**Figures 2A**, **B**). Additionally, decreased expression of CLPTM1L protein in CLPTM1L-shRNA-infected cells was confirmed by Western blot (**Figures 2C**, **D**).

### Functional annotations of CLPTM1L-driven DEGs

**Figure 3:**
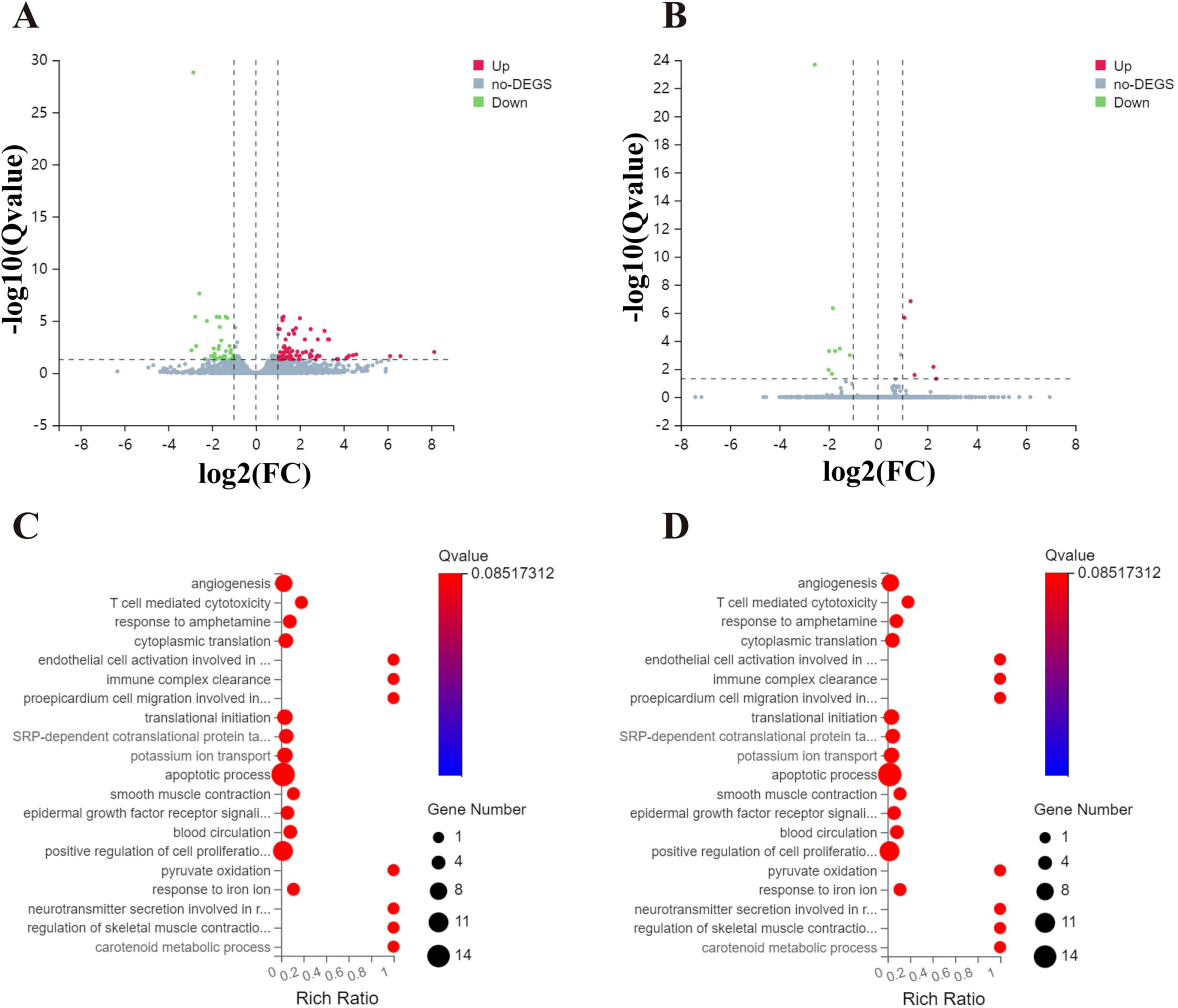
Comparison of gene expression profiles induced by CLPTM1L in HeLa cells. **(A)** Volcano plot illustrating significantly differentially expressed genes (DEGs) between the control-shRNA and CLPTM1L-shRNA1 groups. **(B)** Volcano plot illustrating significantly DEGs between the control-shRNA and CLPTM1L-shRNA2 groups. **(C)** Bubble plot showing the Gene Ontology (GO) functional enrichment analysis of DEGs between the control-shRNA and CLPTM1L-shRNA1 groups. **(D)** Bubble plot displaying the GO functional enrichment analysis of DEGs between the control-shRNA and CLPTM1L-shRNA 2 groups.

RNA-seq analysis was conducted to identify differentially expressed genes (DEGs) driven by CLPTM1L in HeLa cells following knockdown of CLPTM1L expression. Employing thresholds of |Log2 FC| > 1 and Q-value < 0.05, a total of 78 upregulated genes and 50 downregulated genes were identified between the CLPTM1L-shRNA1 and control groups (**Figure 3A**). Similarly, employing the same screening criteria, a total of 14 DEGs, comprising 6 upregulated genes and 8 downregulated genes, were identified between the CLPTM1L-shRNA2 group and the control group (**Figure 3B**). Gene Ontology (GO) enrichment analysis revealed that the differentially enriched genes between the CLPTM1L-shRNA1 and control groups were predominantly associated with biological processes such as angiogenesis, T cell-mediated cytotoxicity, regulation of cell proliferation, and apoptotic processes (**Figure 3C**). Additionally, the differentially enriched genes between the CLPTM1L-shRNA2 and control groups were significantly associated with biological processes such as osteoclast proliferation and retinoid metabolic processes (**Figure 3D**). These functional annotations suggest that knockdown of CLPTM1L expression may contribute to alterations in the proliferation and apoptosis of cervical cancer cells.

### Knockdown of CLPTM1L expression significantly suppressed cell proliferation

**Figure 4:**
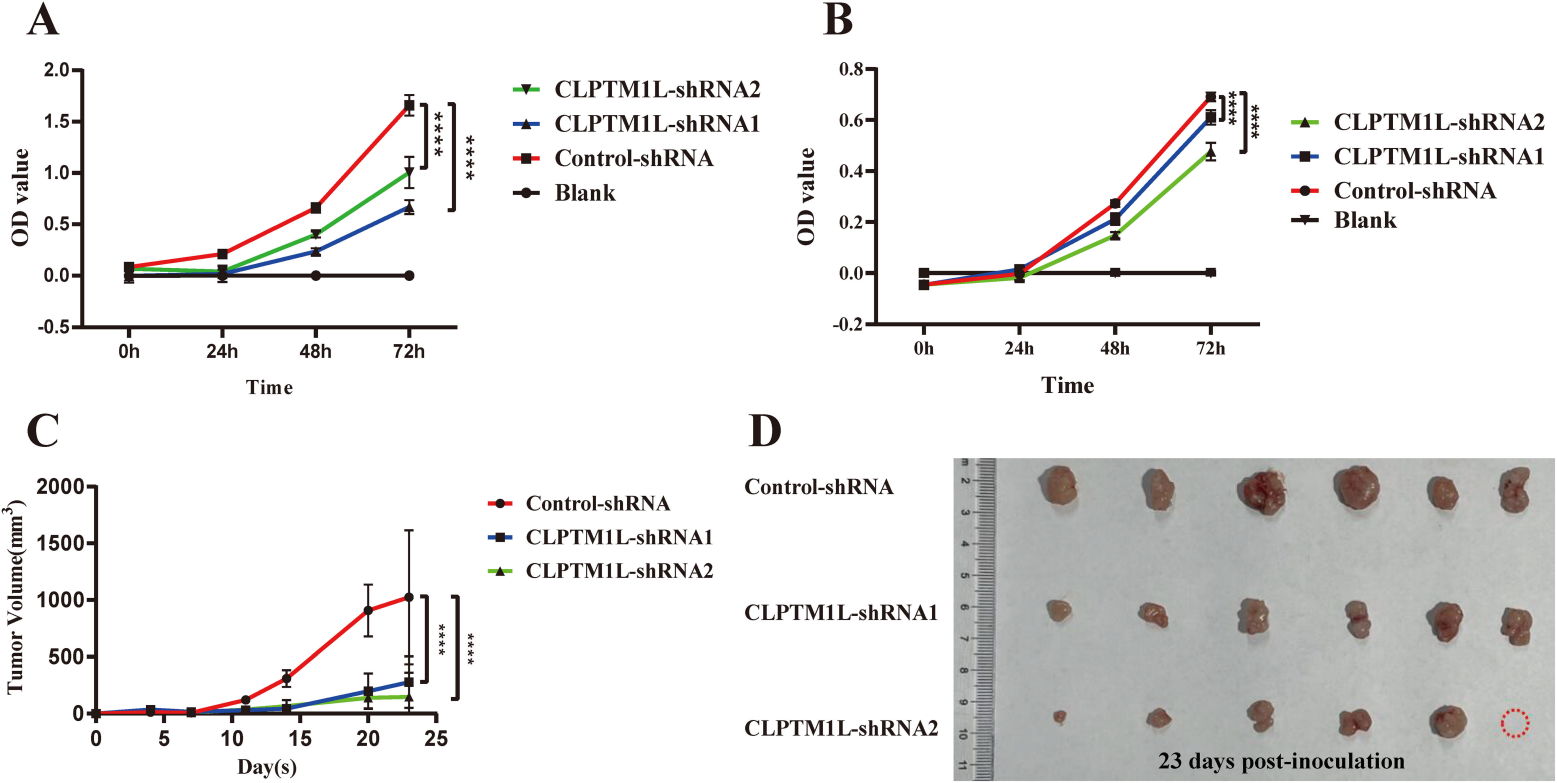
Knockdown of CLPTM1L expression significantly suppressed cervical cancer cell proliferation both *in vitro* and *in vivo*. **(A)** CCK-8 assay demonstrated the effect of CLPTM1L knockdown on the proliferation of HeLa cells. **(B)** CCK-8 assay showed the impact of CLPTM1L knockdown on the proliferation of C-33A cells. **(C)** Tumor growth in HeLa xenograft mice (n = 6/group). **(D)** Images of dissected tumors from HeLa xenograft mice (n = 6/group). ****P<0.0001.

To test the above hypothesis, CCK-8 assay was performed to detect the effect of CLPTM1L on the proliferation of cervical cancer cells. The results demonstrated a notable inhibition in the proliferation ability of HeLa cells in CLPTM1L knockdown groups compared to the control group (**Figure 4A**). Similarly, knockdown of CLPTM1L significantly suppressed the proliferation of C-33A cells compared to the control group (**Figure 4B**). Subsequently, we further validated the role of CLPTM1L in xenograft mouse models. *In vivo* findings revealed that, compared to the control group, the tumor growth rate of nude mice inoculated with CLPTM1L knockdown HeLa cells was significantly reduced (**Figure 4C**), resulting in smaller tumor volumes (**Figure 4D**). Among the six nude mice treated with CLPTM1L-shRNA2 HeLa cell, we observed that only five exhibited detectable tumor tissue. We hypothesized that the lack of a tumor in this specific mouse could be attributed to the inhibitory effect of CLPTM1L-shRNA2 on the proliferation of cervical cancer cells (**Figure 4D**). These results indicate that CLPTM1L can influence the proliferation of cervical cancer cells both *in vitro* and *in vivo*.

### Knockdown of CLPTM1L increased cisplatin-induced cell apoptosis and enhanced cisplatin susceptibility in cervical cancer cells

**Figure 5:**
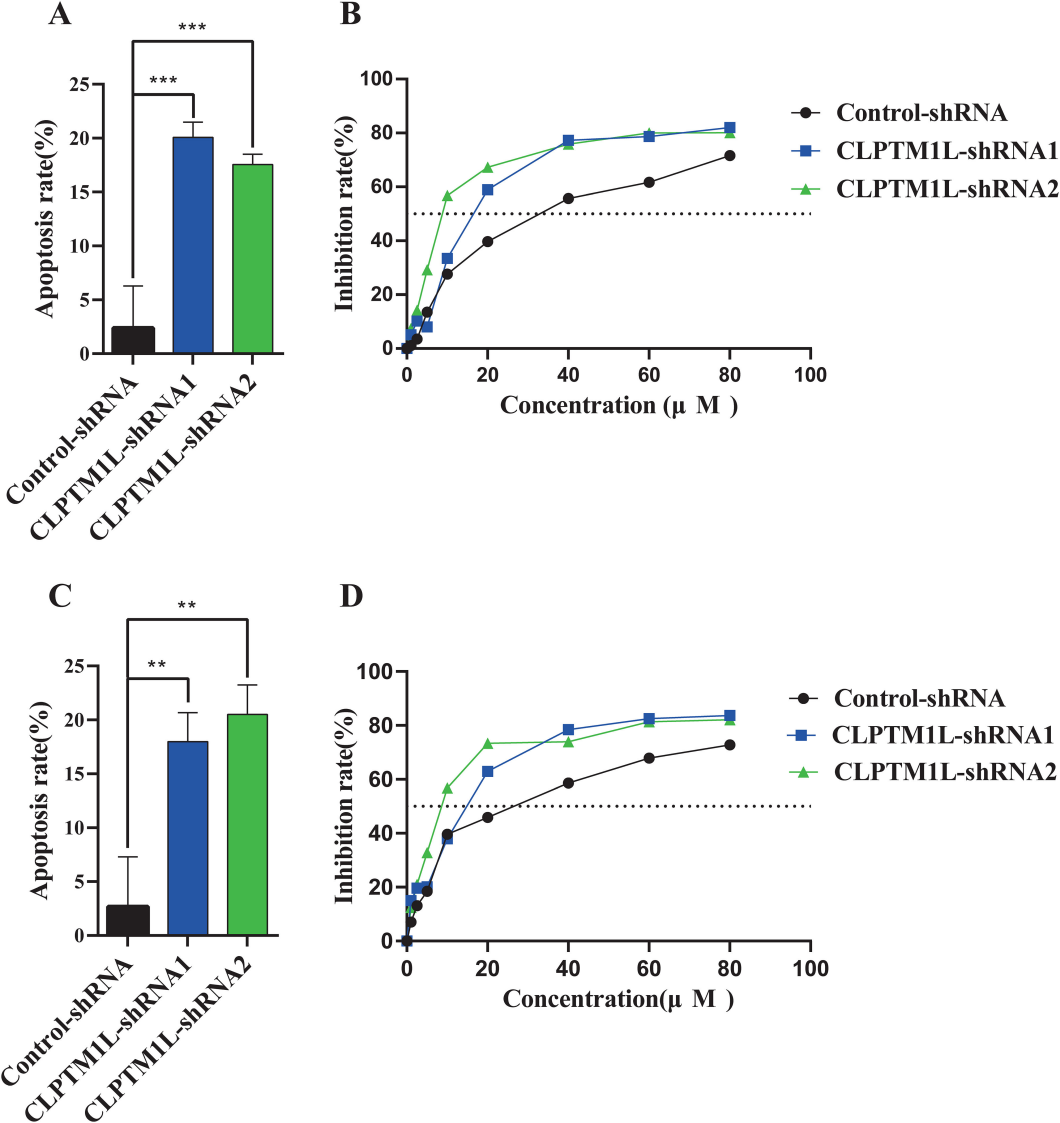
The effect of CLPTM1L knockdown on cisplatin-induced cell apoptosis and the susceptibility of cervical cancer cells to cisplatin. **(A)** Flow cytometric analysis revealed the effect of CLPTM1L knockdown on apoptosis of HeLa cells compared to the control group. **(B)** The growth inhibition rate of CLPTM1L knockdown HeLa cells at increasing concentrations was determined by CCK-8 assay, and the IC50 was calculated. **(C)** Flow cytometric analysis demonstrated the effect of CLPTM1L knockdown on apoptosis of C-33A cells compared to the control group. **(D)** The growth inhibition rate of CLPTM1L knockdown C-33A cells at increasing concentrations was determined by CCK-8 assay, and the IC50 was calculated. **P<0.01, ***P<0.001.

The effect of CLPTM1L on cisplatin-induced cell apoptosis was investigated through flow cytometry analysis following treatment with 30 µM cisplatin. The results revealed a significantly higher apoptosis rate in CLPTM1L knockdown groups compared to the control group in both HeLa and C-33A cells (**Figures 5A**, **C**). To further explore the impact of CLPTM1L on cisplatin sensitivity in cervical cancer cells, Cell Counting Kit-8 assay was conducted to assess cell viability after treatment with varying concentrations of cisplatin. The IC50 of CLPTM1L knockdown cells was markedly decreased compared to the control group (**Figures 5B**, **D**). These findings suggest that knockdown of CLPTM1L enhances cisplatin-induced cell apoptosis and increases cisplatin susceptibility in cervical cancer cells.

### Identification of candidate targets of CLPTM1L

**Figure 6:**
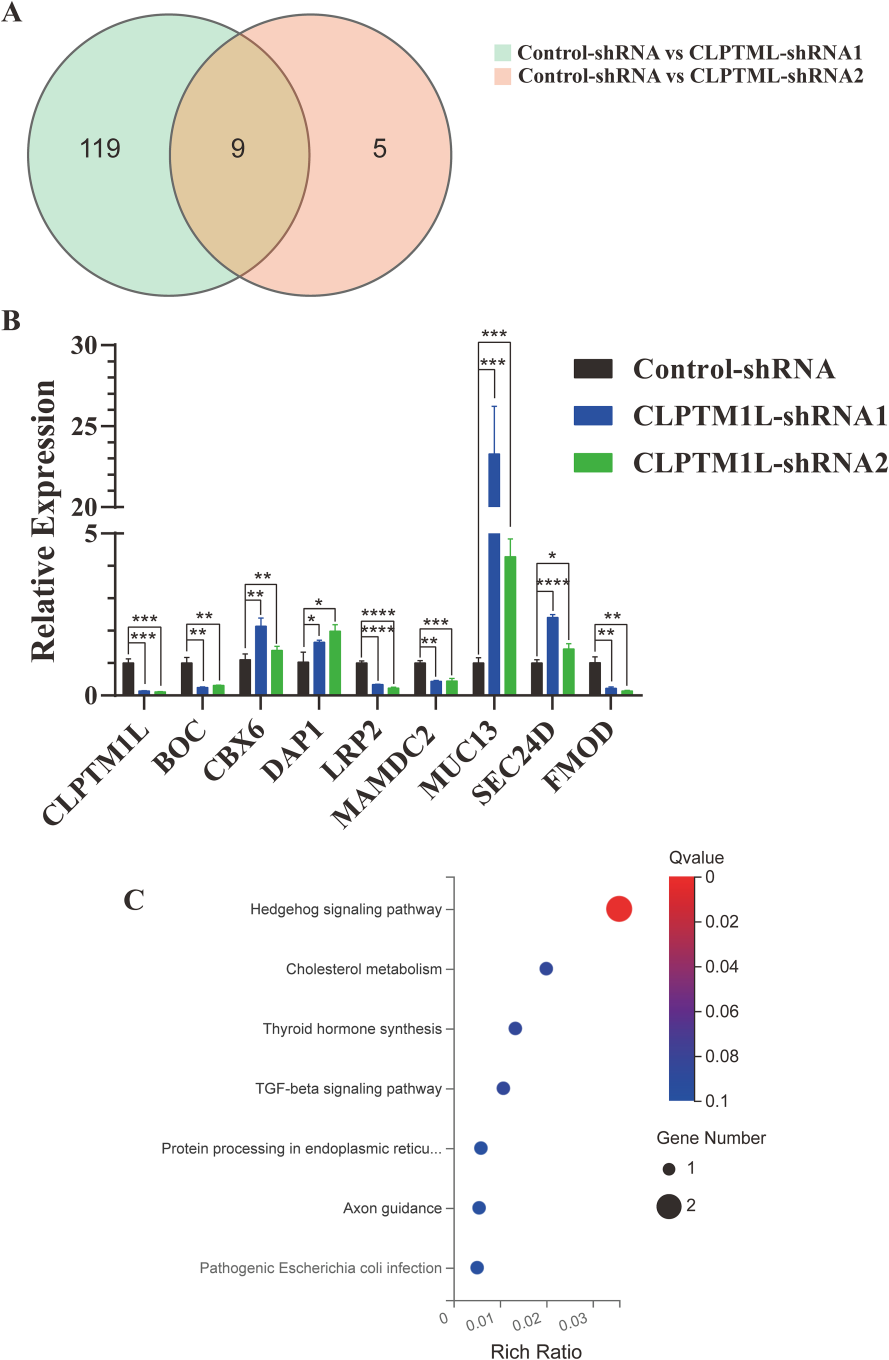
Identification of candidate targets of CLPTM1L. **(A)** Venn diagram depicting 9 overlapping DEGs in the CLPTM1L-shRNA1 group combined with the CLPTM1L-shRNA2 group. **(B)** Real-time PCR confirmed the RNA expression of 9 DEGs in HeLa cells. **(C)** Bubble plots illustrating the top 7 enriched KEGG pathways. *P<0.05, **P<0.01, ***P<0.001, ****P<0.0001.

To elucidate the candidate targets of CLPTM1L, we narrowed down the candidate genes by calculating the intersection of differentially expressed genes from the control-CLPTM1L-shRNA1 and control-CLPTM1L-shRNA2 datasets. The Venn diagram revealed 9 overlapping genes, comprising 4 upregulated genes (DAP1, SEC24D, MUC13, and CBX6) and 5 downregulated genes (CLPTM1L, LRP2, BOC, MAMDC2, and FMOD) (**Figure 6A**). The regulatory effect of these 8 candidate genes by CLPTM1L was confirmed through real-time PCR in HeLa cells(**Figure 6B**). Then the differential genes identified in HeLa cells were further validated in C-33A cells using RT-qPCR. The results indicated that the expression differences of CLPTM1L, BOC, LRP2, DAP1, and SEC24D were consistent with the HeLa cell results, further confirming the reliability of these differential genes’ outcomes (**Supplementary Figure S3**). Additionally, KEGG analysis indicated that the differentially expressed genes are significantly enriched in the Hedgehog signaling pathway (**Figure 6C**).

### Knockdown of BOC or LRP2 inhibits the proliferation of cervical cancer cells

**Figure 7:**
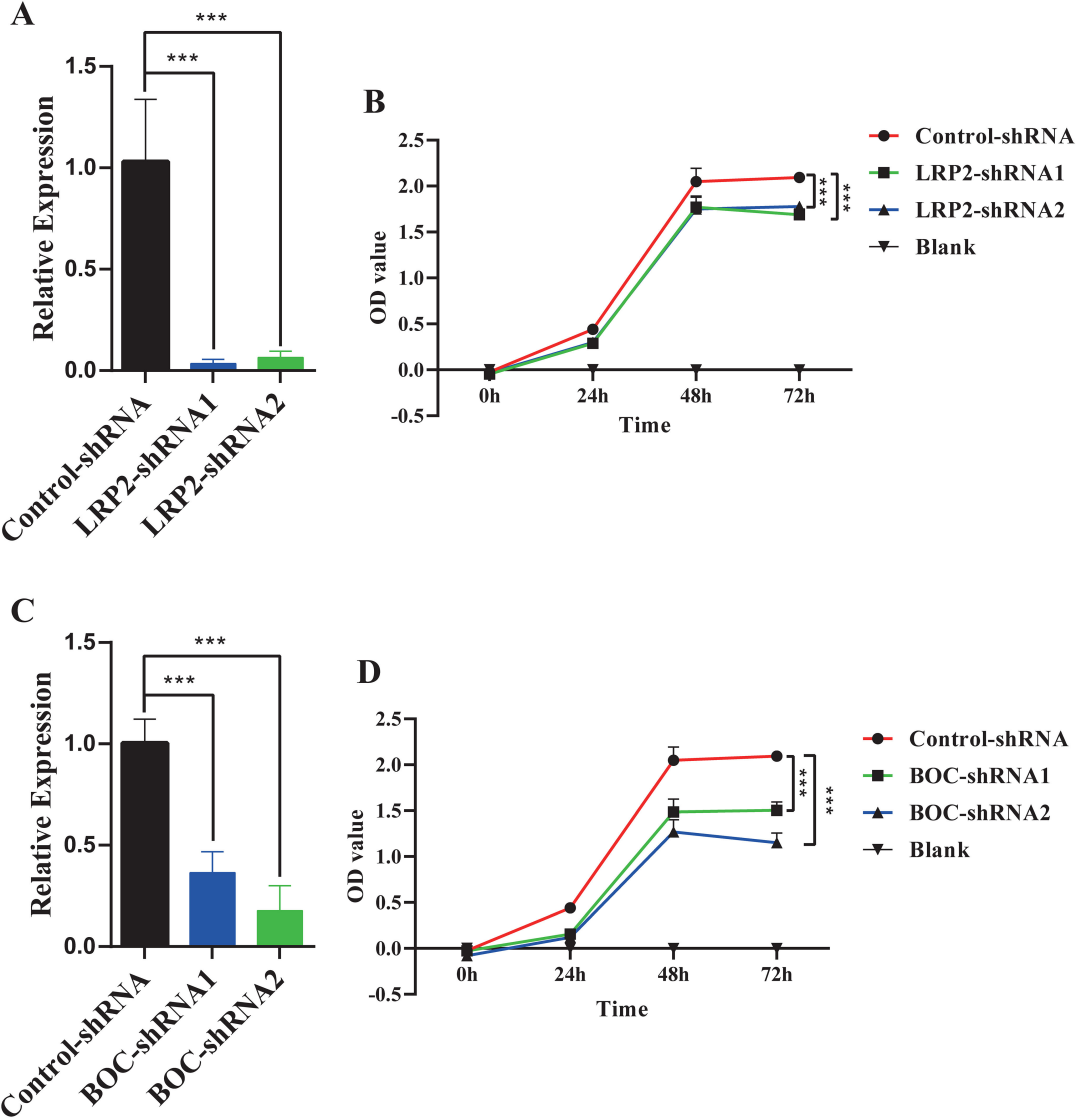
The impact of LRP2 and BOC on the proliferation of HeLa cells. **(A)** RT-qPCR confirmed the knockdown expression of LRP2 in HeLa cells. **(B)** CCK-8 assay demonstrated the effect of LRP2 knockdown on the proliferation of HeLa cells. **(C)** RT-qPCR confirmed the knockdown expression of BOC in HeLa cells. **(D)** CCK-8 assay revealed the effect of BOC knockdown on the proliferation of HeLa cells. ***P<0.001.

To investigate the effects of BOC and LRP2 on the proliferation of cervical cancer cells, lentiviral vectors containing shRNA sequences targeting these genes were utilized to generate HeLa cells with reduced expression of LRP2 or BOC. The decreased expression of LRP2 or BOC was confirmed in the knockdown cells via RT-qPCR (**Figures 7A**, **C**). Subsequently, the impact of LRP2 and BOC on the proliferation of cervical cancer cells was assessed using CCK-8 analysis. The results revealed that, compared with the control group, knockdown of BOC and LRP2 inhibited the proliferation of cervical cancer cells (**Figures 7B**, **D**).

### Knockdown of DAP1 inhibited cisplatin-induced cell apoptosis and reduced cisplatin susceptibility of cervical cancer cells

**Figure 8:**
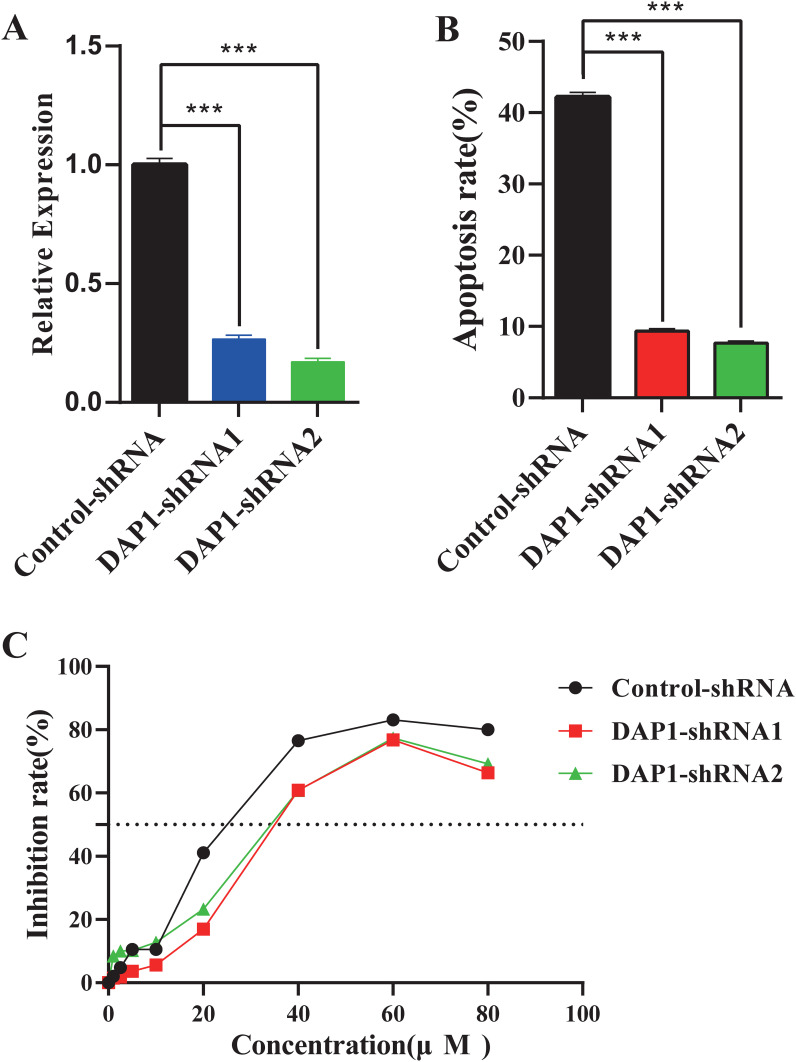
The impact of DAP1 knockdown on cisplatin-induced cell apoptosis and the susceptibility of cervical cancer cells to cisplatin. **(A)** RT-qPCR confirmed the knockdown expression of DAP1 in HeLa cells. **(B)** Flow cytometric analysis demonstrated the effect of DAP1 knockdown on apoptosis of HeLa cells compared to the control group. **(C)** The growth inhibition rate of DAP1 knockdown HeLa cells at increasing concentrations was determined by CCK-8 assay, and the IC50 was calculated. ***P<0.001.

DAP1 has been implicated in tumor cell apoptosis (37, 38), suggesting that CLPTM1L may regulate cisplatin-induced apoptosis and cisplatin sensitivity of cervical cancer cells through DAP1. To test this hypothesis, DAP1 knockdown HeLa cells were generated. The shRNA-mediated knockdown of DAP1 in HeLa cells was confirmed via RT-qPCR (**Figure 8A**). Flow cytometry analysis revealed that shRNA-mediated knockdown of DAP1 inhibited cisplatin-induced apoptosis in HeLa cells compared to the control group (**Figure 8B**). Additionally, the IC50 of DAP1 knockdown cells was significantly higher than that of control cells, indicating that DAP1 knockdown could inhibit the sensitivity of cervical cancer cells to cisplatin (**Figure 8C**).

## Discussion

Recent genetic studies have revealed that the CLPTM1L locus is associated with cervical cancer susceptibility (18–20). However, limited information is available regarding the expression and function of CLPTM1L in cervical cancer. In the current study, we observed a marked increase in the expression of CLPTM1L in cervical cancer cells and tissues. Moreover, genes related to CLPTM1L were found to be enriched in the Hedgehog signaling pathway. Knockdown of CLPTM1L expression significantly suppressed cell proliferation through downstream candidate genes BOC and LRP2. Furthermore, knockdown of CLPTM1L promoted cisplatin-induced cell apoptosis and increased the sensitivity of cervical cancer cells to cisplatin via DAP1.

CLPTM1L expression was markedly increased in cervical cancer cells and tissues compared with ECT1/E6E7 cells and adjacent normal counterparts. Similar findings have been reported in other cancer types, such as lung cancer (29), pancreatic cancer (23), and ovarian cancer (22). Furthermore, a recent study indicated that the expression of CLPTM1L was significantly higher in recurrent patients compared with those in the non-recurrence group (39). These findings suggest that abnormal expression of CLPTM1L may play a crucial role in the pathogenesis and recurrence of cervical cancer.

In this study, we identified 8 differentially expressed genes (DAP1, SEC24D, MUC13, CBX6, LRP2, BOC, MAMDC2, and FMOD) driven by CLPTM1L, with two of them (BOC and LRP2) being enriched in the Hedgehog signaling pathway. The Hedgehog signaling pathway has been shown to play an indispensable role in the growth, invasion, metastasis, recurrence, drug resistance, and radio resistance of cervical cancer (40). However, the potential roles of BOC and LRP2 in cervical cancer remain largely unexplored. Both *in vitro* and *in vivo* results from our study demonstrated that knockdown of CLPTM1L expression significantly suppressed cell proliferation. Previous studies have also reported the role of CLPTM1L in the proliferation of pancreatic cancer cells (23) and oral squamous cells (25). Additionally, we found that knockdown of BOC or LRP2 inhibits the proliferation of cervical cancer cells. BOC expression was upregulated in pancreatic cancer stroma (41), and studies have shown that the Shh-binding protein BOC is upregulated in medulloblastomas and induces granule cell precursor proliferation (42). Furthermore, research has demonstrated that LRP2 is overexpressed in high-grade squamous intraepithelial lesions (HSIL) compared with low-grade squamous intraepithelial lesions (LSIL) (43). Another study indicated that LRP2 is expressed more frequently in melanoma samples compared to nevus samples, and siRNA-mediated knockdown of LRP2 significantly decreases proliferation of melanoma cells (44). These findings are consistent with our results and suggest that CLPTM1L could affect cervical cancer cell proliferation through downstream candidate genes BOC and LRP2.

Our study demonstrated that knockdown of CLPTM1L increased cisplatin-induced cell apoptosis and enhanced the susceptibility of cervical cancer cells to cisplatin. This finding aligns with the role of CLPTM1L in other cancer types, such as lung cancer (29) and ovarian cancer (22, 27). These results further suggest that CLPTM1L may serve as a potential target for sensitizing cervical cancer cells to cisplatin chemotherapy. It is noteworthy that researchers have recently developed human IgG1 anti-CLPTM1L monoclonal antibodies to resensitize ovarian tumor cells to platinum-based drugs (22). In addition, protein agonists or activators developed to target human genes are also increasingly used in the treatment of cervical cancer. For instance, various TLR antagonists/inhibitors have been shown to be effective for the treatment of cervical cancers (45). Therefore, we speculate that in future clinical treatments for cervical cancer, the use of antibodies or inhibitors targeting CLPTM1L could potentially improve the efficacy of cisplatin in treating advanced or recurrent cervical cancer.

Furthermore, our subsequent investigation revealed that shRNA-mediated knockdown of DAP1 inhibited cisplatin-induced cell apoptosis and decreased the susceptibility of cervical cancer cells to cisplatin. A previous study has reported that downregulation of DAP1 expression prevents colorectal tumor cell lines from undergoing apoptosis induced by chemotherapeutic agents, thus contributing to chemotherapy resistance (46). Another study indicated that overexpression of DAP-1 induces apoptosis in the TNF-sensitive L929 fibroblast cell line, as well as in the TNF-resistant U2OS osteosarcoma cell line (47). These findings, combined with our results, suggest that CLPTM1L may influence cisplatin-induced cell apoptosis and cisplatin susceptibility of cervical cancer cells through negative regulation of DAP1.

To the best of our knowledge, this study represents the first exploration into the biological function of CLPTM1L in cervical cancer. Our findings demonstrated a marked increase in CLPTM1L mRNA expression levels in both HeLa and C33A cervical cancer cell lines, as well as in cervical carcinoma tissues, when compared to normal counterparts. This observation paves the way for a deeper exploration of the potential role of CLPTM1L in the pathogenesis of cervical cancer. Importantly, we also demonstrated that the reduction of CLPTM1L expression can enhance the susceptibility of cervical cancer cells to cisplatin-induced apoptosis, a finding that holds significant therapeutic implications. The involvement of the DAP1 gene in this process underscores the complex interplay between CLPTM1L and other key molecular players in the regulation of cervical cancer cell fate.

There are several limitations within our current investigation. Initially, our findings suggest that CLPTM1L may influence cervical cancer cell proliferation through downstream candidate genes BOC and LRP2, while also playing a crucial role in regulating cisplatin-induced apoptosis via DAP1. The intricate mechanisms by which CLPTM1L regulates these downstream genes—BOC, LRP2, and DAP1—require further elucidation in future studies. Additionally, although other CLPTM1L-driven differentially expressed genes are not significantly enriched in specific signaling pathways, further research is warranted to comprehend the functions of these genes in cervical cancer cells. Moreover, while our data indicate a significant increase in CLPTM1L mRNA expression in cervical cancer tumor tissues compared with normal cervical tissues, the differential protein expression of CLPTM1L also necessitates investigation.

## Conclusions

In conclusion, CLPTM1L could impact cervical cancer cell proliferation and cisplatin-induced cell apoptosis, as well as cisplatin susceptibility in cervical cancer cells. This study has provided us with new insights into the development of cervical cancer, highlighting the potential of CLPTM1L as a promising target for enhancing the effectiveness of chemotherapy in the treatment of cervical cancer. However, there is still much work to be done in order to translate scientific research findings into advanced clinical applications.

## Data Availability

The datasets presented in this study can be found in online repositories. The names of the repository/repositories and accession number(s) can be found below: https://www.ncbi.nlm.nih.gov/geo/, GSE267713.
