# Supplementary figures and images for: Cleft lip and palate transmembrane protein 1-like is a putative regulator of tumorigenesis and sensitization of cervical cancer cells to cisplatin
Source: Front Oncol. 2024 Sep 13;14:1440906. doi: 10.3389/fonc.2024.1440906 (PMC11427242; doi:10.3389/fonc.2024.1440906)

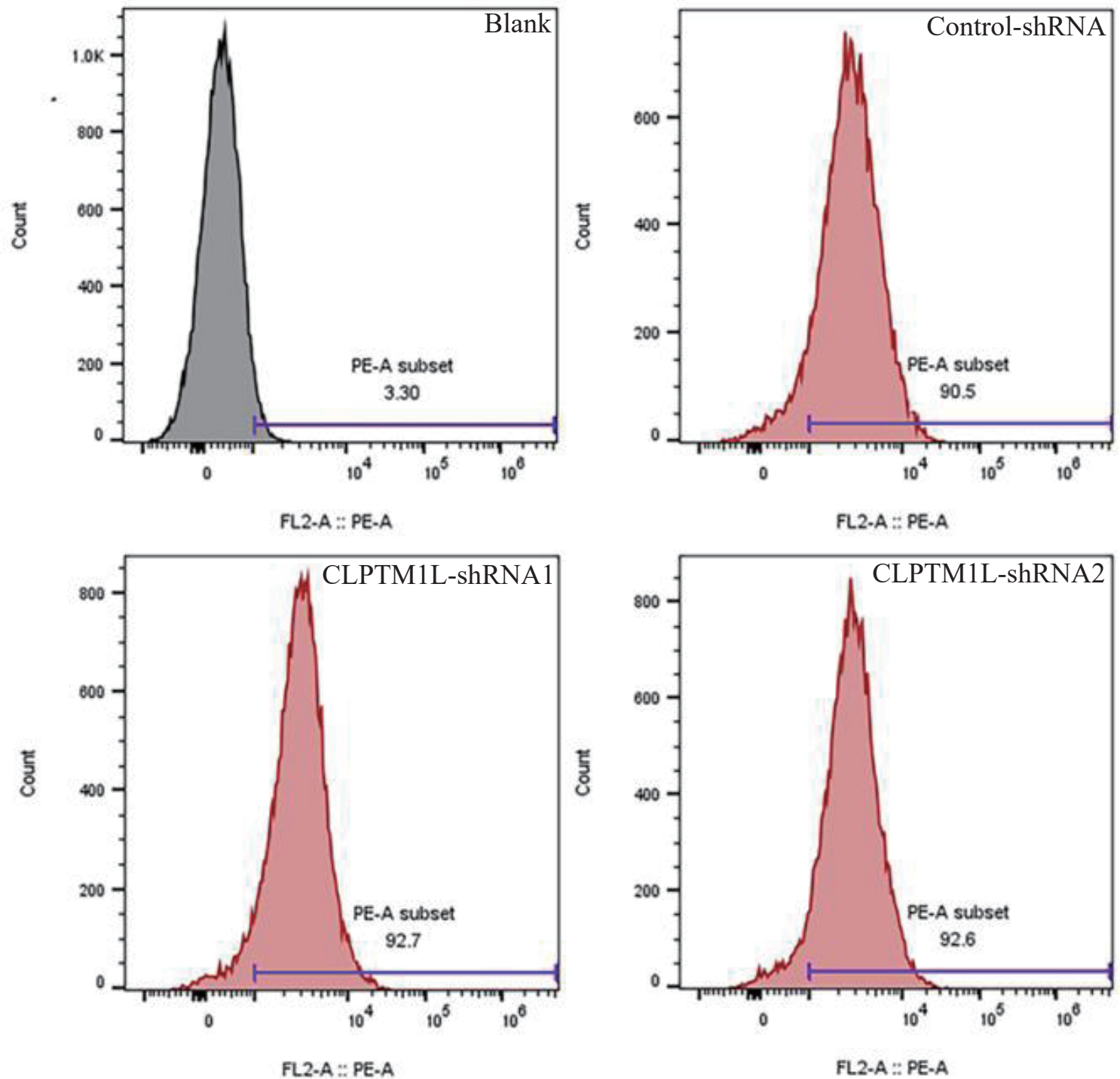

**Figure S1.** Flow cytometric analysis of HeLa cells in the control and CLPTM1L knockdown groups.

Supplement: Supplementary file 1 [file DataSheet1.pdf]

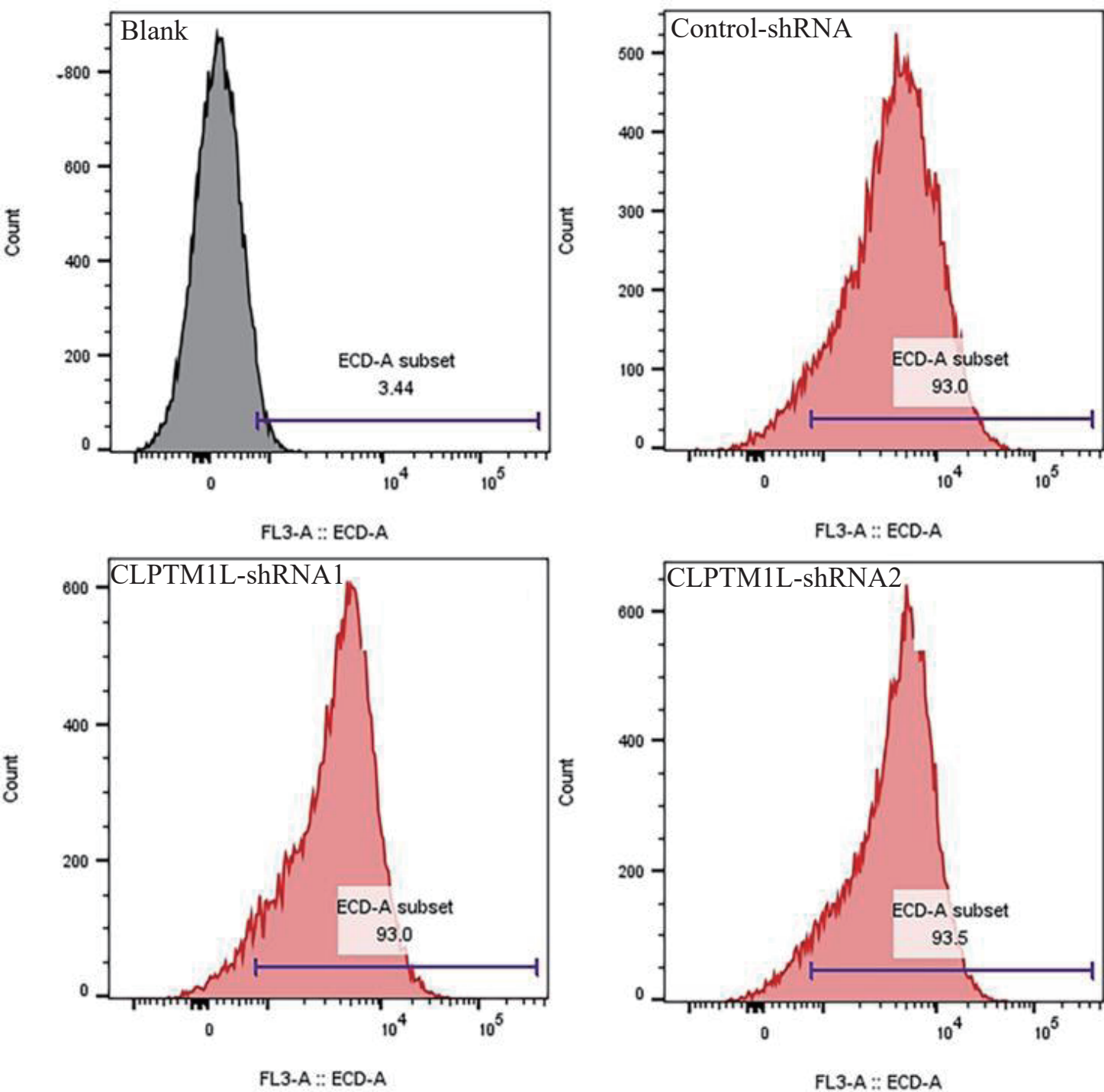

**Figure S2.** Flow cytometric analysis of C-33A cells in the control and CLPTM1L knockdown groups.

Supplement: Supplementary file 2 [file DataSheet2.pdf]

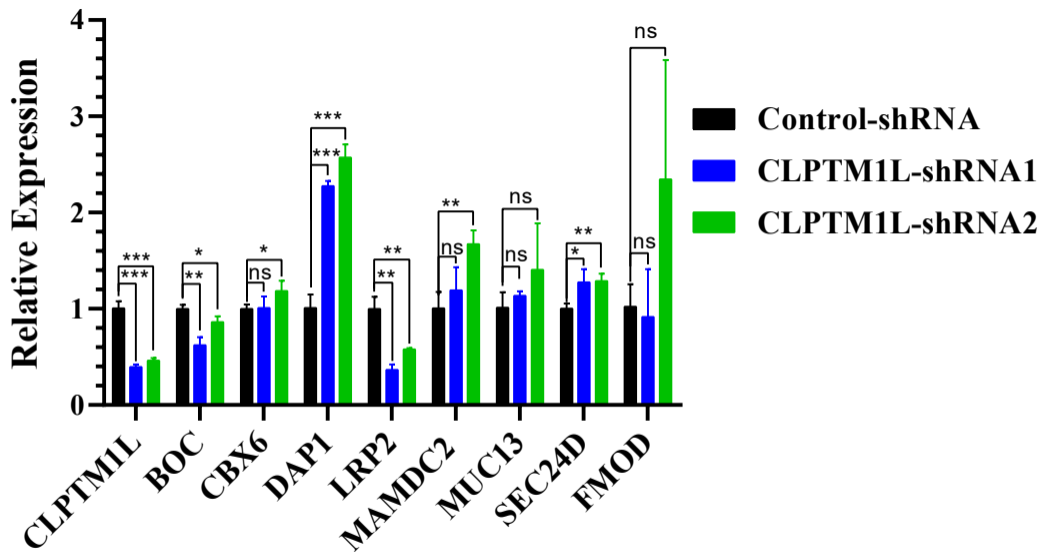

**Figure S3.** Real-time PCR confirmed the RNA expression of 9 DEGs in C-33A cells.

Supplement: Supplementary file 3 [file DataSheet3.pdf]
